# ChatGPT: performance and dermatology specialty certificate examination - Correspondence

**DOI:** 10.1016/j.abd.2023.12.001

**Published:** 2024-02-20

**Authors:** Hinpetch Daungsupawong, Viroj Wiwanitkit

**Affiliations:** a Private Academic Consultant, Phonhong, Vientiane, Lao Democratic People’s Republic; b University Centre for Research & Development Department of Pharmaceutical Sciences, Chandigarh University, Mohali, Punjab, India

Dear Editor,

We would like to respond to a comment on the published article entitled “ChatGPT: performance of artificial intelligence in the dermatology specialty certificate examination”.^1^ The lack of real-time feedback and interaction in ChatGPT could be a vulnerability in the context of the dermatology specialty certificate exam. Even though ChatGPT is capable of giving information and responding to queries based on prior knowledge, it might find it difficult to adjust and pick up on the exam takers’ responses. This constraint might make it more difficult for it to evaluate the clinical judgment and decision-making abilities needed for dermatology. Future developments for ChatGPT in the dermatology specialty certificate exam should concentrate on adding more interactive elements in order to address this weakness. Creating a system that enables a continuous dialogue between the person taking the exam and the AI in order to replicate real-world clinical scenarios is one way to achieve this. ChatGPT could more accurately evaluate the examinee’s capacity to apply dermatological knowledge in a variety of scenarios by actively interacting with them and dynamically modifying its responses in response to their feedback.

Adding to ChatGPT’s educational value would be the implementation of a feedback mechanism that offers explanations and justifications for the AI’s responses. This would facilitate the examinee’s learning process by helping them comprehend not only the right answers but also the logic behind them. The AI’s capacity to adapt and learn from various responses over time may be further enhanced by incorporating machine learning techniques, which would make it a more useful tool for evaluating clinical skills. Additionally, having access to a large and frequently updated database of dermatological cases would be beneficial for ChatGPT. Examinee knowledge would be evaluated more accurately and currently, if the AI took into account a larger variety of cases and integrated the most recent findings and developments in the field. At last, as a fundamental prerequisite for all ChatGPT users, it is now time to establish ethical guidelines for dermatologists regarding the proper and appropriate use of ChatGPT.^2^

## Financial support

None declared.

## Authors’ contributions

Hinpetch Daungsupawong: Ideas, writing, analyzing and approval.

Viroj Wiwanitkit: Ideas, supervision and approval.

## Conflicts of interest

None declared.
